# Recovery from heat, salt and osmotic stress in *Physcomitrella patens* requires a functional small heat shock protein *PpHsp16.4*

**DOI:** 10.1186/1471-2229-13-174

**Published:** 2013-11-05

**Authors:** Cecilia Ruibal, Alexandra Castro, Valentina Carballo, László Szabados, Sabina Vidal

**Affiliations:** 1 Laboratorio de Biología Molecular Vegetal, Facultad de Ciencias, Universidad de la República, Iguá 4225, CP 11400 Montevideo, Uruguay; 2 Institute of Plant Biology, Biological Research Center, Temésvari krt. 62, 6726 Szeged, Hungary; 3 Current address: Whitehead Institute for Biomedical Research, 9 Cambridge Center, Cambridge, MA 02142, USA

**Keywords:** Small heat shock proteins, Osmotic stress, Salinity, *Physcomitrella patens*

## Abstract

**Background:**

Plant small heat shock proteins (sHsps) accumulate in response to various environmental stresses, including heat, drought, salt and oxidative stress. Numerous studies suggest a role for these proteins in stress tolerance by preventing stress-induced protein aggregation as well as by facilitating protein refolding by other chaperones. However, *in vivo* evidence for the involvement of sHsps in tolerance to different stress factors is still missing, mainly due to the lack of appropriate mutants in specific *sHsp* genes.

**Results:**

In this study we characterized the function of a sHsp in abiotic stress tolerance in the moss *Physcomitrella patens*, a model for primitive land plants*.* Using suppression subtractive hybridization, we isolated an abscisic acid-upregulated gene from *P. patens* encoding a 16.4 kDa cytosolic class II sHsp. *PpHsp16.4* was also induced by salicylic acid, dithiothreitol (DTT) and by exposure to various stimuli, including osmotic and salt stress, but not by oxidative stress-inducing compounds. Expression of the gene was maintained upon stress relief, suggesting a role for this protein in the recovery stage. PpHsp16.4 is encoded by two identical genes arranged in tandem in the genome. Targeted disruption of both genes resulted in the inability of plants to recover from heat, salt and osmotic stress. *In vivo* localization studies revealed that PpHsp16.4 localized in cytosolic granules in the vicinity of chloroplasts under non stress conditions, suggesting possible distinct roles for this protein under stress and optimal growth.

**Conclusions:**

We identified a member of the class II sHsp family that showed hormonal and abiotic stress gene regulation. Induction of the gene by DTT treatment suggests that damaged proteins may act as signals for the stress-induction of *PpHsp16.4*. The product of this gene was shown to localize in cytosolic granules near the chloroplasts, suggesting a role for the protein in association with these organelles. Our study provides the first direct genetic evidence for a role of a sHsp in osmotic and salt stress tolerance, and supports a function for this protein particularly during the stress recovery stage of *P. patens*.

## Background

Plants must face and cope with various environmental stresses during their life cycle. Drought, salinity and exposure to extreme temperatures are serious threats to agriculture and have a great impact on plant productivity. Most of these stresses share common consequences as a result from water deprivation, namely osmotic stress and the associated oxidative stress [1].

Tracheophytes have evolved numerous anatomical adaptations to cope with water deficit. These include the presence of vascular tissues, root systems and stomata, which all together help minimizing water loss. By contrast, bryophytes lack these adaptations and must rely on efficient biochemical and physiological mechanisms to survive stress by limiting or repairing the cellular damage resulting from these conditions [2].

Numerous studies have shown that the plant hormone abscisic acid (ABA) plays a crucial role in controlling downstream responses essential for adaptation to abiotic stress. As part of osmotic stress responses, regulation of gene expression occurs in both ABA-dependent and ABA-independent manner [3]. Activation of ABA and stress responsive genes lead to accumulation of proteins belonging to different families, including components of the regulatory networks and proteins implicated in cellular defenses. The latter include proteins such as aquaporins, chaperones, enzymes for osmolyte biosynthesis or detoxification and late embryogenesis abundant (LEA) proteins [4].

Environmental stresses that result in cellular dehydration, such as salt, freezing and water stress lead to similar changes in plant gene expression [5-7]. Disruption of cellular homeostasis induced by exposure to these stresses often causes protein dysfunction. Therefore, cells must employ efficient mechanisms to allow proteins to maintain their functional conformation as well as to prevent the aggregation of denatured proteins under stress. One of the most widespread cellular responses to abiotic stress is the production of heat shock proteins (Hsps). They accumulate in both prokaryotic and eukaryotic cells in response to heat or exposure to various other stress conditions. Strong evidences support a role for Hsps as molecular chaperones, preventing protein aggregation or assisting protein folding during stress [8-12]. Hsps are grouped into five families according to their approximate molecular weight: Hsp100s, Hsp90s, Hsp70s, Hsp60s and Hsp20s, also known as small Hsps (sHsps). Small Hsps belong to a diverse and ubiquitous family of stress proteins that range in size from 12–42 kDa, and are defined by the presence of a conserved C-terminal α-crystallin domain [13]. Plant sHsp protein family is far more complex than that in any other organism investigated to date, probably reflecting a molecular adaptation to stress conditions that are unique to plants [8]. For instance, the *Arabidopsis* genome encodes 19 sHsps which are divided into several subfamilies according to their sequence relatedness and their subcellular localization [8]. Angiosperms have 11 subfamilies that include most but not all of the sHsps. Six of the subfamilies are cytosolic/nuclear localized sHsps (CI-CVI) whereas five others are targeted to organelles: endoplasmic reticulum (ER), peroxisomes (PX), chloroplasts (CP) and mitochondria (MTI and MTII) [8,14,15].

Plant sHsps have been shown to accumulate in response to a broad spectrum of stress factors, such as heat, drought, salinity, low temperature and oxidative stress [8,12,16-21]. Moreover, some members of the cytosolic CI and CII sHsps have been shown to be constitutively expressed in the resurrection plant *Craterostigma plantagineum*, but not in the desiccation-sensitive callus, strongly suggesting a protective role for these proteins during desiccation [19]. Although they are usually not detected under normal growth conditions, several members of the sHsp protein family are developmentally regulated, being the most extensively characterized example of this non-stress regulation during seed development [22]. Developmental regulation of sHsps is generally restricted to some members of the class I and class II sHsps, suggesting that these proteins have distinct regulatory controls during seed maturation as opposed to during stress [23].

Stress-induced expression of sHsps is controlled by heat shock transcription factors (Hsfs) which bind to highly conserved palindromic motifs, so-called heat stress- elements (HSEs) [24-26]. Unlike most other organisms, plant Hsf gene family is highly complex, usually consisting in more than 20 members with high functional diversification [27]. Some members of the Hsf protein family are essential for expression of Hsps during certain developmental stages. This is the case of HsfA9 from *Arabidopsis* which controls accumulation of Hsps during seed maturation. Expression of *Arabidopsis* HsfA9 has been shown to depend on the ABSCISIC ACID INSENSITIVE 3 (ABI3) transcription factor, which regulates various genes during seed desiccation [28]. Furthermore, ABI3 is required for *Arabidopsis* developmental regulation of *Hsp17.4* but is not required for stress induction of this gene [23], reflexing the complexity of the regulatory network that control the expression of this type of genes.

The specific mechanisms by which sHsps confer cell protection are not fully understood. However, in recent years several studies have contributed to develop models showing how these proteins act [15]. Small Hsps have been shown to have the capacity to stabilize and prevent aggregation of non-native proteins via binding through hydrophobic interactions [10,29-33]. Although sHsps do not appear themselves to be able to refold non-native proteins, the current model for sHsps function is that their selective binding to unfolded proteins may facilitate subsequent ATP-dependent refolding by other chaperones [10,29-31,34]. Consistent with this idea, *in vitro* studies of Hsp18.1 from *Pisum sativum* as well as Hsp16.6 from *Synechocystis* sp PCC6803 showed that these proteins bind to unfolded proteins allowing their further refolding by Hsp70/Hsp100 complexes [35].

Although it is generally assumed that sHsps are directly involved in abiotic stress tolerance in plants, much of the information available to date is based on *in vitro* assays, mainly due of the lack of gene-specific knockout mutants in *sHps* genes. *Physcomitrella patens* is an excellent model organism for investigating the role of individual genes by reverse genetics, due to the high frequency of homologous recombination that facilitates the targeted disruption of nuclear genes [36]. In higher plants, disrupted individual genes are usually obtained by screening of random mutants and the probability of altering a specific gene depends very much on the size of the target sequence. Therefore, *P. patens* has significant advantages over other model plants for functional studies of small genes, such as *sHsps,* by gene targeting via homologous recombination. In addition to this, several studies have shown that *P. patens* is highly tolerant to dehydration, salinity, and other abiotic stress factors [37-40], and this tolerance is thought to be based on the mobilization of efficient defense and repair mechanisms in response to stress and during stress relief [38].

This study addressed the question of the function of the duplicated *sHsp* genes *PpHsp16.4* in abiotic stress tolerance in *P. patens*, which was isolated as an ABA-induced gene using suppression subtractive hybridization. *PpHsp16.4* gene product was localized in the cytoplasm and showed to be phylogenetically related to the cytosolic class II family of sHsps. We demonstrated that knockout mutants of the two *PpHsp16.4* genes lead to impaired or delayed recovery of plants from salt, osmotic and heat stress.

## Results

### Identification of an ABA-induced gene encoding a 16.4 kDa sHsp from *Physcomitrella patens*

**Figure 1:**
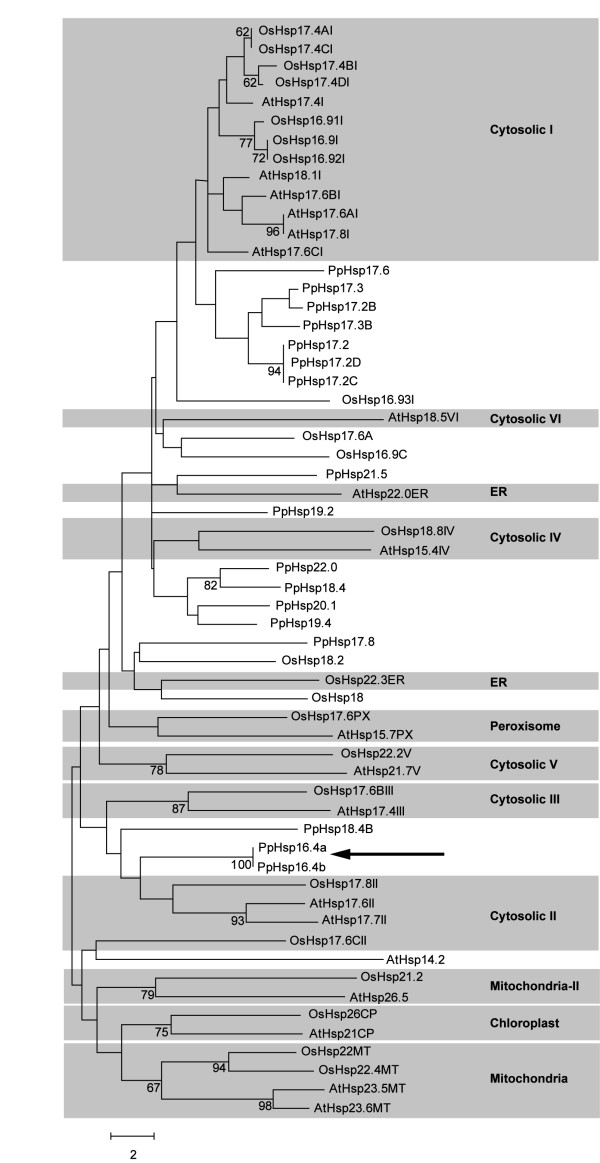
**Phylogenetic analysis of the deduced sHsps of *Arabidopsis*, rice and *P. patens.*** Full-length amino acid sequences were aligned by the CLUSTAL W and a phylogenetic tree was constructed by the neighbor-joining method using MEGA version 5. Accession numbers of the genes from *Arabidopsis* and rice are listed in Methods. *P. patens* sHsps are listed in Table 1. Numbers at branch nodes represent the confidence level of 1000 bootstrap replications. The abbreviations of species are as follows: At: *Arabidopsis thaliana*, Os: *Oryza sativa* and Pp: *Physcomitrella patens*. *Arabidopsis* and rice sHsps are separated into different subclasses which are highlighted in grey squares. ER (endoplasmic reticulum). Arrow shows PpHsp16.4a and PpHsp16.4b from *P. patens.*

In order to identify genes involved in tolerance to abiotic stress in *P. patens*, suppression subtractive hybridization was employed to construct a library enriched in ABA-induced sequences. One of the most abundant sequence in our subtractive library corresponded to a gene encoding a 16.4 kDa sHsp [GenBank: XP_001757324.1]. A search in the *P. patens* full sequence (v1.6) in the public database Phytozome v9.1 (http://www.phytozome.net) [41] showed that *PpHsp16.4* is encoded by two identical nuclear genes, hereby named *PpHsp16.4a* [Phypa_428883] and *PpHsp16.4b* [Phypa_428984]. These genes are 100% identical and exist in a tail-to-tail orientation with 7725 bp separating their stop codons, suggesting their origin from a single ancestral gene that most likely had undergone recent events of tandem duplication and inversion. The conserved α-crystallin domain of sHsps was used as query to search the genome of *P. patens*, revealing the existence of 22 genes encoding sHsps. A list of *P. patens sHsp* genes, their genomic location, and the deduced proteins with their predicted subcellular localization is shown in Table 1. The phylogenetic relationship between the *sHsp* gene family from *P. patens*, *Arabidopsis* and rice was analyzed using ClustalW sequence alignment [42] followed by the neighbor-joining algorithm employing the MEGA 5.05 program [43]. Based on the amino acid sequence homologies, this study clearly placed *PpHsp16.4a* and *PpHsp16.4b* in the same group with the cytosolic class II sHsps (Figure 1).

**Table 1:** **Small Hsp family of *Physcomitrella patens***

**Protein**	**Transcript**	**Phypa_v1.6**	**Phypa_v1.1**	**Scaffold**	**O**	**ORF**	**MW_(kDa)**	**Loc**
PpHsp21.5	Pp1s129_85V6.1	Phypa_444537	Phypa_216427	Scaffold 129:..572968..-..574083	+	190	21.5	nuc/cyto
PpHsp20.1	Pp1s20_253V6.1	Phypa_427505	Pp1s20_253V6.1	Scaffold 20:..1742974..-..1743985	+	180	20.1	cyto
PpHsp19.4	Pp1s350_28V6.1	Phypa_458371	Phypa_199029	Scaffold 350:..287956..-..288477	+	173	19.4	cyto
PpHsp19.2	Pp1s50_96V6.1	Phypa_74946	Phypa_74946	Scaffold 50:..1002205..-..1002857	+	169	19.2	cyto
PpHsp22.0	Pp1s194_15V6.1	Phypa_220338	Phypa_220338	Scaffold 194:..62465..-..63601	-	195	22.0	cyto
PpHsp22.5	Pp1s77_290V6.1	Phypa_438264	Phypa_38520	Scaffold 77:..1524105..-..1525479	+	203	22.5	chlo
PpHsp18.4b	Pp1s144_148V6.1	Phypa_446028	Phypa_138125	Scaffold 144:..1053435..-..1053920	+	161	18.4	cyto
PpHsp18.4a	Pp1s11_289V6.1	Phypa_425098	Phypa_114905	Scaffold 11:..2264806..-..2266292	+	161	18.4	cyto
PpHsp13.4	Pp1s182_60V6.1	Phypa_141732	Phypa_141732	Scaffold 182:..494077..-..494645	+	121	13.4	cyto
PpHsp12.5	Pp1s3_114V6.1	Phypa_64616	Phypa_64616	Scaffold 3:..718664..-..719270	-	111	12.5	nucl
PpHsp16.4b	Pp1s27_332V6.1	Phypa_428984	Phypa_70357	Scaffold 27:..2270773..-..2272356	-	147	16.4	cyto
PpHsp16.4a	Pp1s27_331V6.1	Phypa_428883	Phypa_205434	Scaffold 27:..2261849..-..2263432	+	147	16.4	cyto
PpHsp17.2c	Pp1s85_11V6.1	Phypa_439482	Phypa_213039	Scaffold 85:..60370..-..61284	-	154	17.2	cyto
PpHsp17.3b	Pp1s372_62V6.1	Phypa_459035	Phypa_199515	Scaffold 372: 273630 - 274513	+	154	17.3	cyto
PpHsp17.8	Pp1s380_17V6.1	Phypa_459300	Phypa_37582	Scaffold 380:..166713..-..167180	+	155	17.8	cyto
PpHsp17.6	Pp1s105_133V6.1	Phypa_442034	Phypa_133671	Scaffold 105:..890756..-..891525	+	156	17.6	cyto
PpHsp17.3a	Pp1s8_86V6.1	Phypa_424252	Phypa_174654	Scaffold 8: 747337 - 748527	+	155	17.3	chlo
PpHsp17.2b	Pp1s8_249V6.1	Phypa_424267	Phypa_158998	Scaffold 8:..2505191..-..2506195	-	153	17.2	chlo
PpHsp17.2d	Pp1s8_209V6.1	Phypa_424256	Phypa_65913	Scaffold 8:..2007522..-..2008526	+	153	17.2	chlo
PpHsp17.2a	Pp1s8_244V6.1	Phypa_424313	Phypa_113859	Scaffold 8:..2415925..-..2416902	+	153	17.2	chlo
PpHsp27.5	Pp1s97_106V6.1	Phypa_440946	Phypa_81689	Scaffold 97: 716468 - 718213	+	246	27.5	chlo
PpHsp27.3	Pp1s38_338V6.1	Phypa_72790	Phypa_72790	Scaffold 38: 1916174 - 1917189	+	242	27.3	mito

Protein names were assigned according to their molecular weight (MW) in kiloDaltons (kDa) of the deduced proteins. Transcript name and the *Phypa* number from Phytozome database are listed for the *Phycomitrella patens* genome v1.1 and the genome v1.6. Scaffold values represent the genomic position of the genes. O: orientation, ORF: number of amino acids from the open reading frame, Loc: deduced subcellular localization (nuc: nuclear; cyto: cytosolic; chlo: chloroplastic).

### Expression profile of *PpHsp16.4* gene

**Figure 2:**
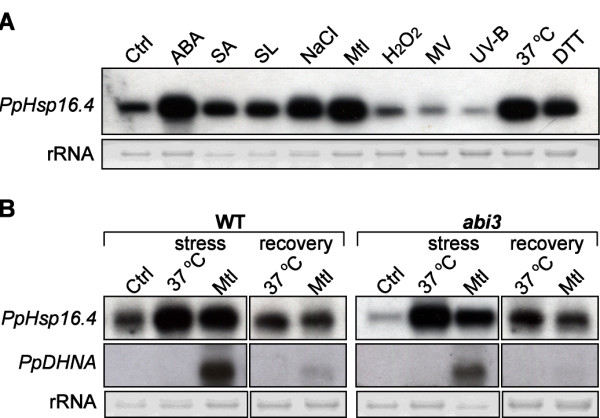
**Expression of *PpHsp16.4*. (A)** Total RNA was extracted from untreated control wild type plants (Ctrl), plants treated for 24 hours with 50 μM ABA, 1 mM SA, 500 mM Mannitol (Mtl), 300 mM NaCl, 10 mM DTT, 100 μM methyl viologen (MV) and 100 μM H_2_O_2_. Samples were also obtained after 24 hours exposure to high temperature (37°C), UVB, and after 2 hours of exposure to strong light (SL). Ten μg of RNA were analyzed by Northern blot using a ^32^P-labeled hybridization probe corresponding to the full-length cDNA sequence of *PpHsp16.4.***(B)** Northern blot analysis of *PpHsp16.4* and *PpDHNA* transcripts in wild type (WT) or *abi3* mutant genotypes. Total RNA was extracted from untreated control plants (Ctrl), plants exposed to 37°C or incubated in medium supplemented with 900 mM mannitol (Mtl). Samples were collected after two days of stress, and 6 hours of recovery. Full -length cDNA sequences of *PpHsp16.4* and *PpDHNA* were ^32^P-labeled and used as hybridization probes. In all experiments, ethidium bromide staining of ribosomal RNA (rRNA) was used to ensure equal loading of RNA samples.

To gain insight into the role of PpHsp16.4 during different stress responses, we monitored transcript levels of the genes encoding this protein in moss gametophyte colonies exposed to different stress conditions or treated with various hormones or chemical compounds producing cellular stress. The genomic sequences of the transcribed regions of *PpHsp16.4a* and *PpHsp16.4b* are identical, including 100% identity in the first 700 bp promoter region, suggesting that the expression of these genes is similar to each other. RNA samples were prepared from controls and from plants treated with the hormones ABA, salicylic acid (SA), and the oxidative stress-inducing compounds H_2_O_2_ and methyl viologen (MV). DTT was included in these experiments as a chemical causing protein misfolding. Plants were also exposed to osmotic stress on mannitol containing plates, to salinity, heat (37°C), UV-B or to strong light conditions. All treatments were performed for 24 h, except for the exposure to strong light, which was done for two hours. Transcript levels of *PpHsp16.4* were analyzed by Northern hybridization using the full length cDNA sequence as a probe (Figure 2A). The detection of a unique hybridization band and the fact that non target sequences corresponding to other *sHsp* genes have generally only short stretches of high sequence identity to *PpHsp16.4*, suggest the lack of cross-hybridization of the cDNA probe to homologous mRNA species. *PpHsp16.4* was found to be constitutively expressed at relatively low levels in control gametophytes. These results were consistent with the microarray based expression data from *P. patens* genes, available at Genevestigator [44] (https://www.genevestigator.com). Analysis of the digital expression profiles of different developmental stages of *P. patens*, showed that *PpHsp16.4* transcripts were expressed at relatively high levels during the gametophyte and sporophyte stages. Although most transcripts of the *sHsp* gene family were found to be present at some level under non stress conditions during specific stages of the plant’s life cycle, only two other transcripts, corresponding to the genes *PpHsp19.2* and *PpHsp18.4b*, were found to be abundant during all developmental stages (Additional file 1), suggesting that these particular members of the *Hsp* gene family may play a role under normal plant growth.

In addition to the observed basal expression in unstressed plants, *PpHsp16.4* was strongly induced by treatment with ABA, SA and DTT and after exposure of plants to heat, strong light, and salt or osmotic stress. In contrast, no increase in mRNA levels of *PpHsp16.4* was observed in response to UV-B or to the oxidative stress inducing compounds, H_2_O_2_ or the herbicide MV, which is a superoxide anion propagator. Similarly, treatment of plants with these compounds for shorter or longer periods of time (4 h and 48 h) failed to induce *PpHsp16.4* gene expression (Additional file 2), supporting that oxidative stress does not play a major role in the regulation of *PpHsp16.4* gene expression. This later results were somewhat unexpected, as most of the CI and CII sHsp genes from other plants species are induced by oxidative stress [15].

Small Hsps have been suggested to be important not only during stress conditions, but also during plant recovery from stress [34]. This prompted us to analyze the expression of *PpHsp16.4* upon relief from heat or osmotic stress (Figure 2B). Plants were incubated at 37°C or in mannitol containing plates for 48 h, and thereafter transferred to optimal growth conditions for 6 h. In these experiments we used higher concentrations of mannitol for imposing a more severe osmotic stress, as these conditions have previously proven to be suitable for the evaluation of the *P. patens* capacity to recover from osmotic stress [37]. Expression of *PpHsp16.4* was compared to the dehydrin *PpDHNA*, which was previously shown to be strongly induced by osmotic stress but rapidly repressed upon stress relief [37,45]. Our results showed that, in contrast to *PpDHNA*, relatively high expression levels of *PpHsp16.4* were still observed after plants returned to optimal conditions, suggesting that *PpHsp16.4* plays a role also during stress recovery.

Recently, Khandelwal et al [46] demonstrated that ABI3 is required for ABA-dependent recovery of *P. patens* from severe dehydration. Stress treatment of *abi3* knockout plants resulted in a small or no reduction of the expression of several genes associated with stress tolerance. However, transcript accumulation of most of the assayed genes was drastically compromised in the *abi3* mutant upon stress relief, suggesting that ABI3 is required primarily for stress recovery. These results prompted us to investigate the biological relevance of ABI3 in the regulation of *PpHsp16.4* expression. We used a null mutant line of *P. patens abi3* which contains no detectable ABI3 due to the disruption of the three copies of the gene [46]. Our results showed that the basal transcript levels of *PpHsp16.4*, observed during normal growth, were considerably reduced in *abi3* compared to the wild type. However, the expression profile of *PpHsp16.4* was very similar in wild type and *abi3* genotypes, both during stress as well as upon stress relief. In contrast, *PpDHNA* transcript accumulation in response to osmotic stress was markedly compromised in the *abi3* mutant, suggesting that different pathways regulate the stress-induced expression of these two genes (Figure 2B). Our results suggest that, in contrast to other stress responsive genes, the stress induction and maintenance of *PpHsp16.4* gene expression in *P. patens* is regulated in an ABI3 independent manner.

### *PpHsp16.4* localized in cytosolic granules

**Figure 3:**
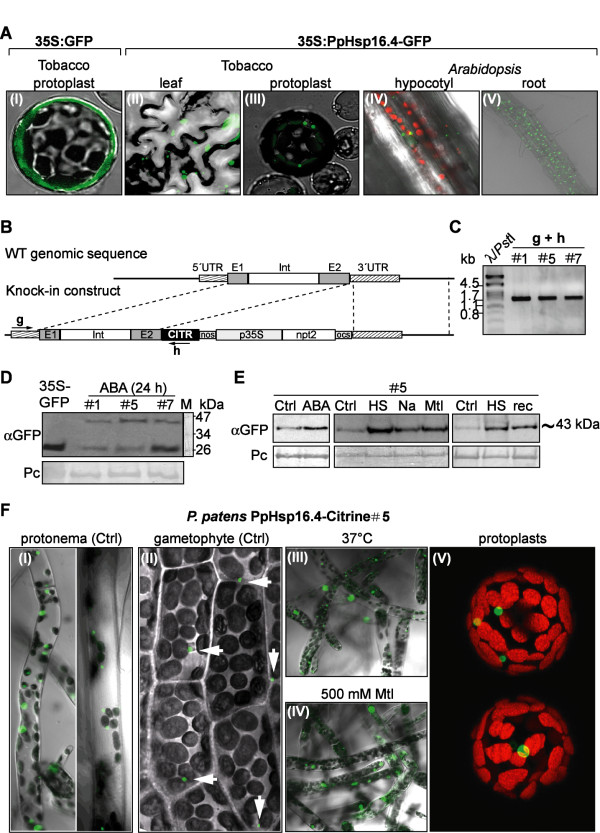
**Subcellular localization of PpHsp16.4. (A)** Confocal microscopy images of: tobacco protoplasts electroporated with 35S:GFP (I) or with 35S:PpHsp16.4-GFP constructs (II); tobacco leaves agroinfiltrated with 35S-PpHsp16.4-GFP construct (III); or *Arabidopsis* transgenic lines overexpressing PpHsp16.4-GFP fusion proteins (IV and V). **(B)** Schematic diagram of *PpHsp16.4* genomic locus and knock-in construct. Exons (E1 and E2), intron (Int), coding sequence of Citrine (CITR), 5’ and 3’ untranslated regions (UTR), are shown. The position of the primers used for PCR analysis of transgenic lines is indicated by arrows. **(C)** PCR analysis of transgenic lines (#1, #5 and #7) **(D)** Immunoblot detection of PpHSP16.4-Citrine fusion proteins in transgenic *P. patens*. Proteins extracted from lines #1, #5 and #7, treated for 24 hours with 50 μM ABA, were analyzed by Western blot using antibodies α-GFP. As a control, a protein sample from *Arabidopsis* expressing GFP was included (35S-GFP). Protein sizes in kDa of the molecular marker (M) are shown. Ponceau red (Pc) staining of Rubisco large subunit was used to ensure equal loading of protein samples **(E)** Immunoblot detection of PpHsp16.4-Citrine *P. patens* . Samples were prepared from controls (Ctrl) or from plants treated with 50 μM ABA or incubated with 300 mM NaCl (Na) or 500 mM mannitol (Mtl) containing plates, or at 37°C (HS: Heat Shock),. Right panel, plants incubated for 48 hours at 37°C (HS) and allowed to recover for 6 hours (rec). **(F)** Spatial regulation and subcellular localization of PpHsp16.4-Citrine fusion proteins transgenic line # 5. Confocal microscopy images of untreated protonema (I), leafy gametophyte (II), protonema exposed for 24 hours at 37°C (III), protonema incubated in 500 mM mannitol supplemented plates (IV) and protoplasts (V). White arrows indicate Citrine fluorescence. Green image: GFP or Citrine emission, in red: chloroplast fluorescence, in gray: transmission light microscopy.

To determine the intracellular localization of *PpHsp16.4* in heterologous cells, the coding region of the gene was fused in frame to GFP and expressed under the control of the constitutive CaMV 35S promoter (35S:*PpHsp16.4*-GFP) in tobacco cells or in transgenic *Arabidopsis* plants (Figure 3A). As a control, a construct constitutively expressing GFP alone was used for transient expression in tobacco protoplasts (Figure 3A-I). Confocal microscopy showed that, while the fluorescent signal of non-fused GFP was found to be homogenously distributed in the cytosol of tobacco protoplasts, *PpHsp16.4*-GFP fusion proteins were present in agroinfiltrated tobacco leaves and transfected protoplasts or in *Arabidopsis* transgenic lines, in round-shaped bodies of different diameters (Figure 3A-II to V). The heterogeneity in the size and the number of fluorescent structures suggested that, rather than targeted to an organelle, *PpHsp16.4* forms large molecular mass structures within the cytoplasm.

To rule out that the observed localization pattern could result from the constitutive overexpression of *PpHsp16.4* in heterologous systems, we examined in detail the targeting of fusion proteins expressed in their natural transcription and translation context in *P. patens*. For that purpose, we generated *P. patens* knock-in lines by inserting the *Citrine* yellow fluorescent protein gene [47] just before the stop codon of *PpHsp16.4* by means of homologous recombination (Figure 3B). In this way, the expression of *PpHsp16.4*-*Citrine* chimeric gene was driven by its native promoter, and the spatiotemporal regulation of the fusion protein could be examined. Gene fusions were confirmed in stable transgenic lines by PCR using primers that recognized a sequence within the *Citrine* gene of the replacement construct combined with primers annealing with the genomic sequences flanking the 5′ region of the two identical *PpHsp16.4a* and *PpHsp16.4b* loci (Figure 3C). We selected three lines that were correctly targeted to the *PpHsp16.4* locus based on the expected size of the PCR amplification products (1787 bp). Accumulation of the fusion protein in ABA-treated transgenic lines was analyzed by Western blot using α-GFP antibodies (Figure 3D). A band of ~43 kDa, consistent with the predicted size of *PpHsp16.4-*Citrine fusion protein was observed in all three lines. An additional band with a molecular mass of 27 kDa was observed in all experiments and corresponded to the cleaved Citrine product. To determine whether the accumulation pattern of PpHsp16.4-Citrine fusion protein correlated with the expression pattern of the wild type *PpHsp16.4* gene, transgenic knock-in lines were analyzed by Western blot for the presence of PpHsp16.4-Citrine after treatment with ABA or in response to various abiotic stress stimuli, including heat (37°C), salt (NaCl) or osmotic stress (mannitol). Accumulation of the fusion protein was also analyzed upon relief from heat stress, by incubating plants at 37°C for 48 hours, and thereafter transferring them to optimal growth conditions for 6 hours. All treatments resulted in higher accumulation of the fusion protein when compared to the controls, indicating that the targeted construct was properly regulated, and that transcript and protein levels of this gene exhibited similar expression patterns (Figure 3E). These transgenic lines were analyzed by confocal microscopy for the determination of the tissue and subcellular localization pattern of the fusion protein. Consistent with the localization analysis in heterologous systems, PpHsp16.4-Citrine accumulated in well-defined regions of the cytoplasm of *P. patens* cells and was missing in other cellular compartments. One or few fluorescent bodies were observed in protoplasts from stable transgenic lines (Figure 3F-V) and in plant cells from different tissues. These structures were found to be always located in the vicinity of chloroplasts when plants were grown under optimal conditions (Figure 3F-I, II). Fluorescent structures were usually more abundant in protonema tissue (Figure 3F-I) than in the leafy gametophyte (Figure 3F-II). Upon exposure to heat or osmotic stress, fluorescent structures became larger and were more abundant than in control cells (Figure 3F-III, IV), resembling the cytosolic granules described by Löw et al [12] in tomato plants exposed to prolonged heat stress.

### Targeted disruption of the two copies of *PpHsp16.4* compromised stress tolerance

**Figure 4:**
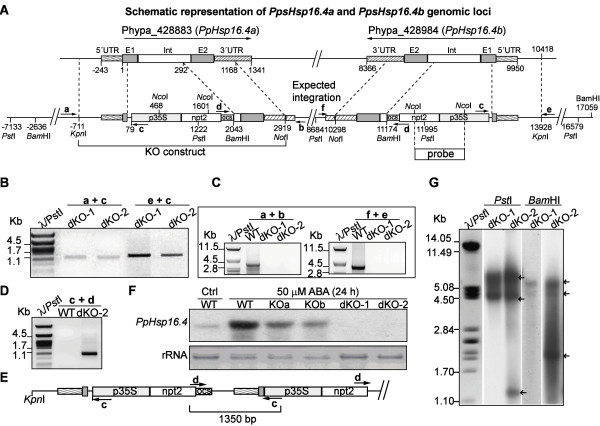
**Targeted insertion at *PpHsp16.4a *and *PpHsp16.4b *loci. (A)** Structure of *PpHsp16.4* genomic loci and expected outcome of construct integration. Exons (E1 and E2) and intron (Int) are boxed. The position of the primers used for PCR analysis of wild type (WT) and mutants is indicated with arrows **(B)** PCR analysis of double knockouts (dKO-1 and dKO-2). Amplification of the 5’ DNA junction sequence for identification of targeting events in *PpHsp16.4a* was done using primer a, located upstream of the construct sequence, in conjunction with primer c, specific for the targeting construct. The 5’region of insertion events in *PpHsp16.4b* was characterized using primer e, specific for the 5’ genomic sequence flanking the construct, together with primer c. The size in kilo base pairs (kb) of the molecular weight marker (λ/*Pst*I) is shown to the left. **(C)** PCR amplification of the genomic sequence of *PpHsp16.4* loci*.* Amplification of *PpHsp16.4a* was done using primers a and b, specific for the flanking 5’and 3’sequences respectively. Amplification of *PpHsp16.4b,* was done using the external primers f and e. **(D)** Detection of construct concatemers using primers c and d, specific for the targeting construct. **(E)** Schematic representation of a partial head-to-tail structure of concatenated DNA revealed by sequencing of the PCR product obtained in **(D)**. **(F)** Northern blot analysis of WT, single knockout lines (KOa, KOb), and double knockout lines. Total RNA isolated from plants treated with 50 μM ABA for 24 h or control (Ctrl) was hybridized to the radiolabeled full-length cDNA sequence of *PpHsp16.4.* Ethidium bromide staining of ribosomal RNA (rRNA) was used to ensure equal loading of RNA samples. **(G)** Southern blot analysis of WT, dKO-1 and dKO-2 genotypes. Genomic DNA was digested with *Pst*I and *Bam*HI, and hybridized with a radiolabeled DNA probe shown in **(A)**. Arrows indicate hybridization bands.

To assess the function of *PpHsp16.4* in stress tolerance, we used gene targeting to generate disruption mutants of *PpHsp16.4* genes. As both genes are 100% identical at the nucleotide level, a single knockout construct was used for gene replacement of *PpHsp16.4a* and *PpHsp16.4b* by homologous recombination (Figure 4A). In this construct, most of the first exon and the intron of the target genes were replaced by a kanamycin selection cassette (*nptII*). Gene targeting events were identified by PCR to select lines in which insertion of the transgene had occurred either in one of the two genes, or in both genes in the same transformation event (Figure 4B). To identify insertion events in *PpHsp16.4a*, we used a reverse primer located within the selection cassette (primer c) together with a forward primer (primer a), located outside the gene targeting construct in a genomic region upstream of *PpHsp16.4a*, that has no homology to sequences upstream of *PpHsp16.4b*. Similarly, to identify insertion events within the *PpHsp16.4b* gene, we used a forward primer located within the selection cassette (primer c) together with a reverse primer (primer e) that binds a genomic region outside the construct, downstream of this gene. The expected sizes of the PCR products that would originate from specific gene targeting events at the loci *PpHsp16.4a* or *PpHsp16.4b* is of 1353 bp or 1378 bp, respectively.

Several lines were obtained in which either *PpHsp16.4a* or *PpHsp16.4b* had been targeted (data not shown). Two independent lines (dKO-1 and dKO-2) were identified, in which homologous recombination had occurred at the 5′end of both *PpHsp16.4a* and *PpHsp16.4b* loci (Figure 4B). To analyze whether these lines had incorporated the construct by two events of homologous recombination, primers were designed to recognize specific sequences outside the construct, downstream of *PpHsp16.4a* (primer b) or of *PpHsp16.4b* (primer f). When these primers were used in combination with a primer located within the selection cassette (primer d), no PCR product was observed in any of the lines analyzed. This result could be indicative of an integration pattern derived from a HR event at one end and a non-homologous end joining event at the other. To investigate whether a wild type copy of the targeted locus remained adjacent to the inserted construct, we used the primers that recognize genomic sequences flanking the construct of either *PpHsp16.4a* (primers a and b) or *PpHsp16.4b* (primers f and e). PCR products from the expected sizes (3882 bp *PpHsp16.4a* and 4024 bp for *PpHsp16.*4b) were obtained from the wild type genotype, but no amplification was observed when using DNA from any of the dKO lines, indicating the absence of a full wild type copy of these genes in the double mutants (Figure 4C).

Complex integration patterns, derived from concatenation of DNA or from other possibilities, have been shown to occur frequently when transforming *P. patens* genome with a targeting vector [48]. To analyze the nature of the DNA integration within *PpHsp16.4a* and *PpHsp16.4b* loci in the double targeted lines, we used two outward-pointing primers (c and d) specific to the selection cassette to identify possible head-to-tail concatemers. A single PCR fragment of 1.3 kb was observed for the dKO-2 line (Figure 4D). This fragment was cloned and sequenced and the results support a model where at least two copies of the construct DNA had integrated in a head-to-tail orientation, but with the loss of the 3′ *PpHsp16.4* genomic sequence. No PCR product was obtained when using a single primer only, indicating the absence of head-to-head or tail-to-tail full length concatemers (data not shown). A schematic representation of a possible integration pattern in either *PpHsp16.4a* or *PpHsp16.4b* loci in dKO-2, is shown in Figure 4E. To determine whether the construct had integrated only at *PpHsp16.4* loci or additional copies were introduced in other genomic locations, we performed Southern blot using part of the selection cassette as a probe (Figure 4G). Genomic DNA from the double KOs was digested with *Pst*I or with *Bam*HI restriction enzymes, which cleave within the selection cassette, at the positions shown in Figure 4A. If a single insertion had occurred in each of the target genes, four hybridization bands of 8.38, 4.58, 7.7 and 3.3 kb should be detected after *Pst*I digestion, and two bands of 5.4 and 4.7 kb, after *Bam*HI cleavage. The restriction pattern obtained with *Pst*I resulted in two bands of 8.4 and 4.5 kb in both mutants. It is possible that the 7.7 and 3.3 kb bands were not detected due to the short area of coverage of the probe. In case of the dKO-2 line, an additional band of 1.5 kb was observed, supporting multiple integration events in the same locus. Digestion with *Bam*HI produced two hybridization bands of 5.4 and 4.7 kb in both KOs and an additional band of 1 kb was observed in dKO-2. These results suggest that no additional inserts had been integrated in other genomic regions outside of *PpHsp16.4a* and *PpHsp16.4b* loci in the double mutants, although a complex integration pattern, most likely involving the deletion of part of the DNA lying between both genes, had occurred for at least dKO-2 line.

**Figure 5:**
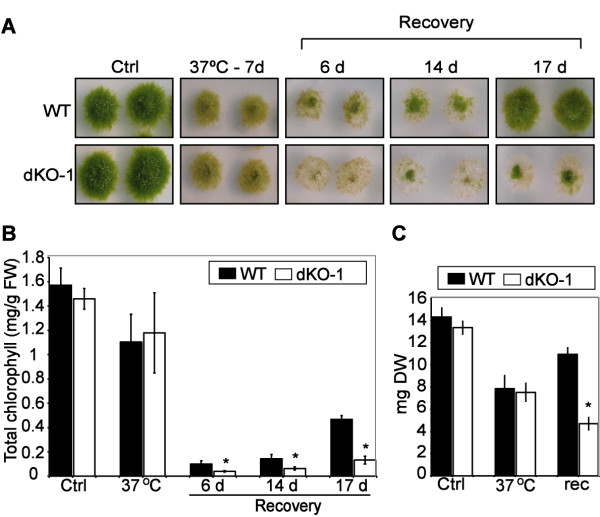
**Effect of heat stress on *P. patens *wild type and double knockout mutant. (A)** Phenotypic comparison of WT and dKO-1 plants grown in optimal conditions (Ctrl) and incubated at 37°C for 7 days. Plant colonies were subsequently transferred to normal medium, and the stress recovery process was monitored during time and photographed at 6, 14 and 17 days. **(B)** Total chlorophyll content (mg/g/fresh weight) per colony was determined from non-stressed controls (Ctrl), heat stressed plants for 7 days (37°C) and at the indicated time points of recovery in standard growth medium. **(C)** Dry weight per colony (mg) of control (Ctl), stressed (37°C) and plants allowed to recover in optimal growth conditions for 17 (rec). The values shown are means from one representative technical replicate. Error bars indicate SD (n = 15). Three biological replicates were carried out. Significant differences of at least 0.05 confidence level between the wild-type and the KO lines are marked by *.

**Figure 6:**
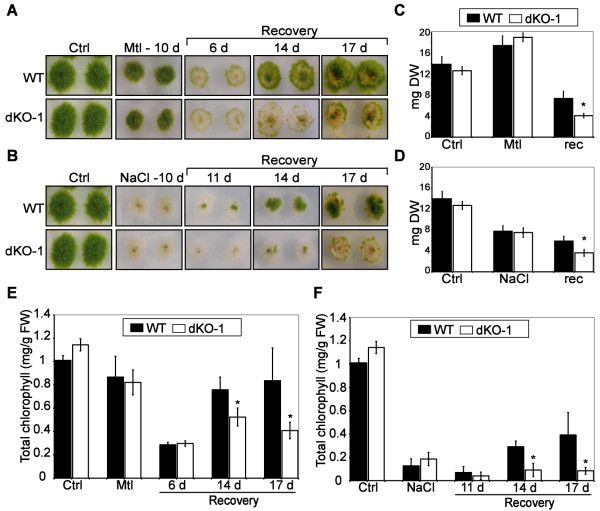
**Effect of osmotic and salt stress on *P. patens *wild type and knockout mutant. (A)** Photograph images of WT and dKO-1 lines grown in optimal conditions (Ctrl), incubated for 10 days in plates supplemented with 900 mM mannitol (Mtl-10 d), and during 6, 14 and 17 days of recovery from osmotic stress. (**B)** Same as above but employing 500 mM NaCl as stressor and 11, 14 and 17 days of recovery. **(C)** Dry weight per colony in milligrams (mg) of controls (Ctl), plants stressed with 900 mM Mannitol for 10 days (Mtl) and plants allowed to recover from stress for 17 days (rec). **(D)** Same as **(C)** but using 500 mM NaCl as stressor. **(E)** Total chlorophyll content (mg/g/fresh weight) per colony from non-stressed controls (Ctrl), mannitol stressed plants (Mtl) and from plants allowed to recover from stress at the indicated time points in days. **(F)** Same as above but using NaCl as stressor. The values shown are means from one representative technical replicate. Error bars indicate SD (n = 15). Three biological replicates were carried out. Significant differences of at least 0.05 confidence level between the wild-type and the KO lines are marked by *.

To confirm the loss of function of *PpHsp16.4* in the KO lines, transcript accumulation of the *PpHsp16.4* genes was analyzed by Northern blot after ABA-treatment of wild-type and mutant genotypes. *PpHsp16.4* transcript was lower in the single knockout mutants (KOa and KOb) than in wild-type plants and was completely eliminated in the double KO lines (Figure 4F). Single KOs (a and b) and the double KOs 1 and 2 lines were analyzed for phenotypic alterations during normal growth or stress conditions. In all conditions assayed, the phenotype of the two independent single disruption lines did not differed significantly from the wild type plants. Also, both double mutants were phenotypically indistinguishable from each other in our experiments and therefore only the data of dKO-1 is shown in Figures 5 and 6. No phenotypic changes in growth rate or in developmental progression were observed in the single or in the double knockout mutants in standard growth conditions (Figure 5A, Ctrl). To obtain functional data on the role of *PpHsp16.4* during stress conditions, we monitored growth and chlorophyll content of wild type and mutant lines exposed to various stress factors. No differences in the sensitivity to oxidative stress-inducing compounds, such as H_2_O_2_ and MV, were observed between the wild type and the mutant lines (data not shown). In contrast, the double KO lines were unable to recover from prolonged heat (Figure 5) or from severe salt (0.5 M NaCl) or osmotic (0.9 M mannitol) stress (Figures 6A and 6B). Whilst wild type plants usually displayed full recovery 17 days after returning to optimal growth conditions, little or no growth took place in the double KO lines. Under these conditions, chlorophyll content and dry weight of the double mutant was reduced by 50% when compared to the wild type plants. These results showed that PpHsp16.4 has an essential role in the recovery of *P. patens* from heat and from prolonged or severe salt and osmotic stress conditions.

## Discussion

### Abiotic stress-induction of *PpHsp16.4* is independent of ROS and ABI3

This study provides genetic evidence for the involvement of a sHsp from *P. patens* in heat, salt and osmotic stress tolerance. *PpHsp16.4* gene, which was found to be abundant in a subtractive library enriched in ABA-induced cDNAs, encodes a 16.4 kDa protein belonging to the cytosolic class II subfamily of plant sHsps. *PpHsp16.4* was shown to be expressed at low levels under optimal growth conditions, and up-regulated in response to exposure to various abiotic stress factors, including strong light, heat, salt and osmotic stress. These stresses are usually accompanied by oxidative cell damage due to the accumulation of ROS, and therefore, it has been suggested that these compounds play an important role in *sHsp* gene regulation [49]. Consistent with this, many of the CI or CII sHsp encoding genes from *Arabidopsis* and from other angiosperms, are expressed in response to oxidative stress among a broad spectrum of other stressors [15,22,50-53]. Furthermore, heat shock transcription factors have been shown to sense ROS and in turn activate *Hsp* gene expression [54,55], and some data suggest that Hsps protect cells against ROS [20,56]. However, in our experiments, expression of *PpHsp16.4* gene was not affected by H_2_O_2_ or by the herbicide methyl viologen, indicating that ROS are not the primary signals for the induction of this particular gene. In contrast, induction of the unfolded protein response by treatment of plants with DTT resulted in a strong accumulation of *PpHsp16.4* transcripts, suggesting that rather than ROS themselves, the damaged proteins may act as signals for gene induction, as it has been previously suggested [57].

In addition to stress, *PpHsp16.4* was induced by treatment with ABA and SA, two phytohormones that have been linked to heat stress signaling and basal thermotolerance in plants [58,59]. SA has been shown to induce the expression of *sHsp* genes from *Arabidopsis*[60,61] and *P. patens*[62], suggesting a conservation of this regulatory pathway between angiosperms and mosses. Elevated levels of both ABA and SA have been measured in various plant species in response to heat stress [58,60,63], although their role in the regulation of *Hsp* gene expression during heat stress is not clear [59,64].

A recent report demonstrated that PpABI3 was essential during the plant’s recovery from stress for maintenance of transcripts encoding proteins that are critical for tolerance [46]. The high expression levels of *PpHsp16.4* observed when plants were transferred to optimal conditions after exposure to stress, suggests a role for the corresponding protein in the recovery stage. However, expression of *PpHsp16.4* during stress and upon stress relief was ABI3-independent. Nevertheless, the basal expression of the gene under optimal growth conditions was reduced in *abi3* mutant, suggesting that different regulatory pathways operate in the regulation of *PpHsp16.4* and supporting distinct developmental and stress regulation pathways of sHsps, as previously suggested [23]. These results also suggest that PpHsp16.4 may be involved in cellular functions under non-stress conditions as it has been suggested for several members of the plant *sHsp* gene family that exhibit constitutive expression under non-stress conditions in various developmental stages [51,65].

### A functional *PpHsp16.4* gene is required for recovery from heat, salt and osmotic stress

Elucidation of plant sHsp function *in vivo* has been challenging due to the limited T-DNA insertion lines that are available in *Arabidopsis* to facilitate the analysis of sHsp deficient plants. The possibility to carry out targeted gene disruption in *P. patens* by means of homologous recombination, allowed us to assess the role of *PpHsp16.4* genes in stress tolerance, by generating single and double knockout mutants of these genes. Our results showed that although wild type plants normally exhibit a low but constitutive expression level of *PpHsp16.4* under optimal growth, we were not able to detect any significant difference in growth or morphology between the wild type and any of the mutant genotypes during non-stress conditions. Moreover, no phenotypical differences in the stress response were observed between the wild type and the single and double knockout mutants when growing in the presence of high salt or osmotic stress, or after incubation at 37°C. Nevertheless, we were able to detect a clear phenotype in the double knockout plants at the stress recovery stage. When plants exposed to heat, salt or osmotic stress were transferred to optimal growth medium, the double knockout lines were affected or failed completely to resume growth. Our data is consistent with other studies showing that *P. patens*, like other bryophytes, survives stress by employing molecular mechanisms that protect cellular integrity during stress and enable damage repair upon stress relief allowing plants to resume growth [37,39,46]. In this context, in accordance to the current models for sHsp action [15], PpHsp16.4 may contribute to stress tolerance by preventing stress-induced irreversible protein aggregation and, together with other chaperones, help re-solubilizing aggregated proteins allowing cells to return to equilibrium during recovery. It is intriguing that such phenotypic alterations were observed in spite of the large number of *sHsp* genes present in *P. patens* and the possible redundancy in their function.

### Subcellular localization of PpHsp16.4 reveals possible distinct roles for this protein during stress and under physiological conditions

The generation of *P. patens* knock-in lines expressing the Citrine fluorescent protein fused to the C-terminal end of PpHsp16.4, allowed us to examine the subcellular localization and the spatiotemporal regulation of the fusion protein, when driven by the native promoter of the gene. PpHSP16.4 was expressed in all tissues under normal growth conditions as shown by the presence of single or multiple fluorescent bodies within the cytoplasm of the cells. Interestingly, these structures were always located in the vicinity of chloroplasts, suggesting a possible role for this protein in association with chloroplast functions. Chloroplasts are the primary targets of damage caused by high light, which interferes with oxygenic photosynthesis, a phenomenon known as photoinhibition [66], and exposure of plants to excess light strongly induced *PpHsp16.4* transcript accumulation. Moreover, a recent report has demonstrated that a cytosolic class I *Arabidopsis* Hsp17.8 plays a role under standard physiological conditions, in targeting proteins to the outer membranes of chloroplasts [67]. These authors showed that in non-stressed cells, *Arabidopsis* sHsp17.8 acts as an ankyrin repeat protein 2A (AKR2A) cofactor and binds chloroplasts as a dimer assisting protein targeting to this organelle. However, under heat shock conditions, expression of the gene was strongly induced and Hsp17.8 was converted to high oligomeric forms, as shown for other sHsps. Using heterologous tobacco and *Arabidopsis* transient or stable expression systems, we showed that PpHsp16.4 localized in the cytoplasm under the form of large and shapeless structures, resembling the heat-induced granules described by Löw et al [12]. The observed fluorescent structures probably represent oligomeric complexes of PpHsp16.4-GFP fusion proteins, as it is well known that plant sHsps form oligomers of different orders [15,68]. The expression profile of *PpHsp16.4* in *P. patens* supported a similar pattern as for the *Arabidopsis* sHsp17.8. Indeed, upon heat shock or osmotic stress, the expression of *PpHsp16.4* was strongly induced both at the transcript and the protein level, and the cytosolic signals of the fusion protein were converted to large structures which are consistent with high oligomeric protein complexes. This supports the idea that when acting as chaperones in the stress response, PpHsp16.4 could bind to unfolded proteins in large complexes, thereby preventing them from forming nonspecific aggregates, as it has been proposed for sHsp function [33].

## Conclusions

Since the development of *P. patens* as a model system for reverse genetics by using homologous recombination technologies, numerous studies have contributed to genetically dissect plant responses to environmental stress. Despite the significant amount of data concerning the structure, gene regulation and function of the plant *sHsp* gene family, attributing specific roles to individual proteins in stress tolerance has been difficult. Using this excellent model, we provide the first direct genetic evidence for a role of a sHsp in osmotic and salt stress tolerance. Our results support a function of this protein particularly during the stress recovery stage of *P. patens*, emphasizing the importance of cellular mechanisms that protect protein integrity and enable damage repair upon stress relief. Our results also suggest a role for this sHsp under non stress conditions, in association with chloroplasts, as it has been shown for some class I sHsps from *Arabidopsis*.

## Methods

### Plant material, growth conditions and stress treatments

*Physcomitrella patens* Grandsen wild type [69] was used for all experiments described in this study. The *abi3* triple knockout mutant [46] was kindly provided by Prof. Ralph S. Quatrano. Plants were grown and maintained axenically on cellophane overlaid BCDAT medium (1.6 g L^-1^ Hoagland’s 1 mM MgSO_4_, 1.8 mM KH_2_PO_4_ pH 6.5, 10 mM KNO_3_, 45 μM FeSO_4_, 1 mM CaCl_2_, 5 mM ammonium tartrate and 10 g L^-1^ agar) as described by Ashton and Cove [70]. To generate protonemal cultures plant material was macerated with a sterile mortar and pestle in 2 ml of sterile double distilled water. For micropropagation, moss colonies were cut with a scalpel and plant fragments were transferred to fresh medium with cellophane. All plants were grown at 22°C under a photoperiod of 16 hours light, with a photon flux of 60 μmol m^-2^ sec^-1^. Three weeks-old colonies were used for all experiments. Abscisic acid, H_2_O_2_, methyl viologen, salicylic acid, dithiothreitol, NaCl and mannitol treatments were incorporated in the BCDAT medium. The concentrations of these compounds are indicated in the figure legends. Strong light treatment was performed by exposing the colonies with a photon flux of 350 μmol m^-2^ sec^-1^. UV-B treatment was done using a Hi-Tech lamp (G25T8E, Japan) with UV-B 0.034 mW/cm^2^. All treatments were performed for 24 hours except for strong light that was performed for 2 hours. All experiments were repeated at least three times. The different plant genotypes were grown together in the same plate, five colonies per genotype, and five plates per treatment and time point.

Tobacco plants of *Nicotiana tabacum* cv Petit Havana [71] was used for transient expression analysis. For protoplast preparation, plants were grown from surface-sterilized seeds in Murashige and Skoog medium [72] and 2% sucrose at 22°C with a 16 h day length at a light irradiance of 200 μmol m^-2^ sec^-1^. For agroinfiltration experiments, soil-grown tobacco plants were used.

*Arabidopsis thaliana* (Col-0) was used for stable transformation and confocal microscopy. For *in vitro* growth, seeds were surface sterilized for 15 min in 7% of bleach with 0.05% Tween-20, washed with sterile water, incubated at 4°C for 3 days and plated in Petri dishes with half strength MS medium (2.4 g L^-1^ Murashige and Skoog, 5 g L^-1^ sucrose, 0.5 g L^-1^ Monohydrate 2- ethanesulfonic acid and 10% agar). Plants were grown at 22°C with a photoperiod of 16 h light and a photon flux of 120 μmol m^-2^ sec^-1^.

### Subtracted cDNA library construction

Moss colonies were treated with 20 μM ABA during 6, 24 and 48 hours and total RNA was extracted from untreated controls and ABA treated plants. Fifty μg of total RNA extracted from each time point were pooled together to constitute a single ABA treated sample and an untreated control. Two μg of mRNA purified from the RNA samples were used for cDNA synthesis. The Clontech PCR Select-cDNA Subtraction Kit (BD Biosciences Clontech) was employed for suppressive subtractive hybridization, using the samples derived from ABA-treated plants as tester and the controls as driver. The secondary PCR products were purified and inserted into pCR II vector and transformed into *Escherichia coli* TOP 10 competent cells, using TA cloning kit Dual Promoter from Life Technologies. Approximately 800 clones were selected for insert sequencing.

### Phylogenetic analysis

Translated protein sequences from *Arabidopsis* and rice *sHsp* genes were retrieved from Phytozome database, based on previous analysis reported in the literature [8,65]. For the identification of *sHsps* genes in the genome of *P. patens*, all annotated genes in the Phytozome database were screened for the presence of the conserved α-crystalline domain, using this sequence as a query for a BlastP search. Sequences were aligned with ClustalW in a MEGA version 5 software [42,43] for subsequent phylogenetic analysis. Construction of phylogenetic trees was done using the Neighbor joining method. Due to the large differences in the sizes of some members of the *P. patens* sHsp protein family, only 17 of the 22 genes from this species are shown in the results represented in Figure 1.

Accession numbers of *sHsp* genes from rice were: Os16.9 I [Os01g0136100], Os16.91 I [Os01g0136000], Os16.92 I[Os01g0136200], Os16.93I [Os01g0135900], Os17.4A I [Os03g0266900], Os17.4B I[Os03g0267200], Os17.4C I [Os03g0267000], Os17.4D I [Os03g0266300], Os17.6C II[Os02g0217900], Os17.8II [Os01g0184100], Os17.6B III [Os02g0782500], Os18.8 IV [Os07g0517100], Os22.2 V [Os05g0500500], Os17.6 PX [Os06g0253100], Os22.3 ER [Os04g0445100], Os26 CP [Os03g0245800], Os22 MT [Os02g0758000], Os22.4 MT [Os06g0219500], Os16.9C [Os02g0711300], Os17.6A [Os01g0135800], Os18 [Os11g0244200], Os18.2 [Os02g128000], Os21.2 [Os02g0107100]. Accession numbers of *sHsp* genes from *Arabidopsis* were: AtHsp17.4I [At3g46230], AtHsp18.1I [At5g59720], AtHsp17.6BI [At2g29500], AtHsp17.6AI [At1g59860], AtHsp17.8I [At1g07400], AtHsp17.6CI [At1g53540], AtHsp18.5VI [At2g19310], AtHsp22.0ER [At4g10250], AtHsp15.4IV [At4g21870], AtHsp15.7PX [At5g37670], AtHsp21.7 V [At5g54660], AtHsp17.4III [At1g54050], AtHsp17.6II [At5g12020], AtHsp17.7II [At5g12030], AtHsp14.2 [At5g47600], AtHsp26.5 [At1g52560], AtHsp21CP [At4g27670], AtHsp23.5MT [At5g51440], AtHsp23.6MT [At4g25200]. Accession numbers from the deduced sHsp proteins of *P. patens* are listed in Table 1.

### Construct design for targeted gene disruption of *PpHsp16.4* in *P. patens*

The construct for disruption of *PpHsp16.4* was done using the vector pUBW302 [37], containing the *nptII* gene driven by the constitutive 35S promoter and the 3′UTR of the *ocs* gene. A 792 bp genomic fragment from the 5′region of the *PpHsp16.4* gene (bases -711 to 79 bases from the start codon) was cloned upstream from the 35S promoter, whereas a 876 bp DNA fragment corresponding to bases 292 to 1168 of the genomic sequence of the gene was inserted downstream of the *ocs* terminator signal. The genomic sequence for the 5′ insertion was PCR amplified using gene-specific primers containing sequences for restriction enzymes *Kpn*I (forward primer “ccggtacccatccattgatctaac”) and *Hind*III (reverse primer “cacaagcttctgggactgtggat”) to facilitate the subsequent cloning of the fragment. The 3′sequence of the gene was PCR amplified from genomic DNA using the forward primer “tgtggatccggactaccaattgtgact” and reverse primer “acagcggccgcactagcacctcccaa”, and cloned in pUBW302 vector using *Bam*HI and *Not*I restriction enzymes.

### Construct design for PpHsp16.4-Citrine *in vivo* fusion in *P. patens*

For the knock-in gene fusion construct we used the vector pCTRN-NPTII 2, generated by Makino H. et al. and acquired from the Physcobase clone collection (http://moss.nibb.ac.jp). A DNA fragment corresponding to bases -25 to 967 (from the start codon) of the genomic sequence of *PpHsp16.4*, including the entire coding sequence of the gene and lacking the stop codon, was PCR amplified from *P. patens* genomic DNA using the primers “gtggtaccttggtcaacttgagagaa” (forward) and “atcgatatctccccccatagtcacctc” (reverse), containing *Kpn*I and *EcoR*V restriction sites, respectively. This fragment was cloned upstream and in frame with the *Citrine* gene of the vector pCTRN-NPTII 2. Subsequently, a 1131 bp 3′region of *PpHsp16.4* genomic locus, including the 3′UTR of the gene and part of the adjacent genomic sequence (bases 995 to 2099), was amplified by PCR using the primers “acaggatccgggctctctagaaatgac” (forward) and “ttcgcggccgcaagcttttggtttatg” (reverse), which contained restriction sites for *Bam*HI and *Not*I, respectively. This fragment was inserted downstream of the *nptII* selection cassette of the vector in order to direct two events of homologous recombination in the *PpHsp16.4* locus.

### Generation of *P. patens* transgenic lines

The generation of moss protoplasts and the subsequent transformation were done as described by Schaefer et al [69]. Briefly, isolated protoplasts (final concentration of 1.6x10^6^ per ml) were incubated with 30 μg of linearized plasmid DNA (digested with *Kpn*I). After polyethylene glycol treatment, protoplasts were incubated for 7 days at 22°C on BCDAT medium supplemented with 10 mM CaCl_2_ and 0.44 M mannitol. Protoplasts were thereafter transferred to BCDAT medium supplemented with 40 μg mL^-1^ of G418 and cultured for 10 days. Protoplasts were subsequently allowed to grow for 10 days on BCDAT medium without selection and finally returned to BCDAT medium containing 40 μg mL^-1^ of G418. Plants showing growth after two weeks on selection medium were analyzed for the correct incorporation of the transgene on the *PpHsp16.4* locus.

### Molecular characterization of knockout mutants

The incorporation of the gene targeting construct in the *PpHsp16.4* locus was confirmed by PCR amplification of genomic DNA using the following primers: (a) “ataaaaacaaataaatacaaaaacct” or (e) “catccattctacttgttgaaccacct”, forward primers for *PpHsp16.4a* or *PpHsp16.4b*, respectively, in combination with the reverse primer (c) “ctttctctgtgttcttgatgcagttag”. To identify possible head-to-tail concatemers we used primer (c) together with primer (d) “ctacccgtgatattgctgaagagc”. Full lenght genomic sequences were amplified by using primer (a) with primer (b) “tatagatattccttatattcaactcaa“ for *PpHsp16.4a*, and primer (e) together with (f) “catcttcttgcattattcttggggg” for *PpHsp16.4b* gene. To verify the lack of functional *PpHsp16.4a* and *PpHsp16.4b* genes due to targeted gene disruption in the *P. patens* mutants, total RNA was isolated from wild-type and mutant genotypes, treated with 50 μM ABA for 24 hours. Northern blot analysis were performed as described below in this section, using the full length cDNA sequence of *PpHsp16.4* radiolabeled as a probe.

### Northern blot

Total RNA was isolated from control or treated *P. patens* tissues corresponding to 20 to 30 colonies, using standard procedures based on phenol/chloroform extraction followed by LiCl precipitation. Ten μg of total RNA separated in denaturing formaldehyde agarose gels were transferred to nylon membranes (Hybond XL, Amersham Pharmacia Biotech), according to Sambrook et al [73]. Membranes were prehybridized at 65°C in 5× SSPE, 5× Denhardt’s solution, 0.2% SDS and 0.5 mg mL^-1^ denatured salmon sperm DNA. Hybridizations were performed at 65°C overnight. The DNA fragments corresponding to full-length cDNA sequences of *PpHsp16.4* or *PpDHNA* [EMBL:AAR13080.1 or Phypa_221321] were labeled with [α^32^P]-dCTP using the Rediprime II random priming labeling system (Amersham Pharmacia Biotech) and used as probe in these studies. Filters were washed twice for 30 min at 65°C with 5× SSC-0.5% SDS, and twice using the same conditions with 1× SSC-0.5% SDS, and exposed in autoradiography films. Ethidium bromide staining was used to ensure equal amounts of loading of RNA in the samples.

### Southern blot

Genomic DNA was extracted as described by Dellaporta et al [74] with an additional RNase treatment and phenol extraction using fresh plant material. The genomic DNA was analyzed by digesting 10 μg with *Bam*HI and *Pst*I restriction enzymes. Restricted DNA was separated in 1% agarose gels and transferred into nylon filters (Hybond XL, Amersham Pharmacia Biotech) according to Sambrook et al [73]. Membranes were prehybridized and hybridized as described for the Northern blot analysis. A DNA fragment corresponding to an *Nco*I restriction fragment consisting on part of the 35S promoter and the *nptII* gene from the selection cassette, was labeled with [α^32^P]-dCTP using the Rediprime II random priming labeling system (Amersham Pharmacia Biotech) and used as probe in these studies.

### Molecular characterization of *P. patens* PpHsp16.4-Citrine knock-in lines

The incorporation of the gene targeting construct *PpHsp16.4-Citrine* in the *PpHsp16.4* locus was confirmed by PCR amplification of genomic DNA using the following primers: (g) “ccggtacccatccattgatctaac” and (h) “cgccctcgccggacacgctgaact”, specific for a genomic region upstream the targeting construct and the Citrine gene, respectively. To verify the correct expression of *PpHsp16.4:Citrine* in *P. patens* knock-in lines, Western blots were performed with protein samples extracted from transgenic lines untreated controls, or treated with 50 μM ABA, incubated at 37°C or in 300 mM NaCl or 500 mM mannitol containing plates for 24 hours. Furthermore, plants were incubated for 48 h, at 37°C and thereafter transferred to optimal growth conditions for 6 hours, to determine the fusion protein levels during stress recovery. Western blot analysis were performed as described in Saavedra et al [37]. Briefly, soluble plant proteins were extracted in 50 mM Tris–HCl pH 7.2, 250 mM sucrose, 5 mM EDTA pH 8.0, 10 mM β-mercaptoethanol, 10 mM MgCl_2_, 1 mM CaCl_2_ and 1 mM PMSF. Ten μg of soluble proteins were separated in 10% polyacrylamide gels and electroblotted onto nitrocellulose membranes (Hybond-ECL, Amersham, GE Healthcare). Blots were incubated with 0.5% Ponceau red for 10 minutes and washed with distilled water. Ponceau staining of ribulose-1,5-bis-phosphate carboxylase/oxygenase (Rubisco) large subunit served as loading controls. Membranes were blocked in Tris-buffered saline (TBS, 20 mM Tris–HCl, 150 mM NaCl pH 7.4) containing 5% (weight in volume, w/v) skimmed milk powder and 0.2% Tween-20 for one hour at room temperature. The primary antibodies used in this study were the commercial anti-GFP antibody produced in rabbit (Sigma Aldrich), 1 mg/ml stock, diluted 1/4000 in TBS, 0.1% Tween, or the polyclonal antisera anti-PpDHNA [37], diluted 1/1500. Horseradish peroxidase-labeled goat anti-rabbit antibody (Sigma Aldrich) diluted 1/10000 in TBS-Tween 0.1% was used as secondary antibody. Protein reactions were visualized in autoradiography films using the ECL detection system.

### Construct design for cDNA fusion with GFP

Full length cDNA of *PpHsp16.4*, lacking the stop codon, was PCR amplified from total ARN samples extracted from *P. patens* treated with 50 μM ABA for 24 hours, using the following primers: forward “gtggatccttggtcaacttgagag” and reverse “atatctcgagtgctttccccccatagtcac”. The resulting PCR fragment was cloned using *Bam*HI and *Xho*I restriction enzymes into the pENTR2B entry vector (Gateway, Invitrogen). This construct was thereafter used for LR-mediated recombination of *PpHsp16.4* cDNA sequence into the pK7FWG2 destination binary vector [75], containing the GFP coding sequence under the regulation of the 35S promoter. The *PpHsp16.4* cDNA sequence was fused in frame to the 5’ end of GFP, resulting in the chimeric gene 35S:*PpHsp16.4*-GFP. The resulting construct was introduced in *Agrobacterium tumefaciens* strain pGV3101/pMP90 [76] by electroporation.

### *Arabidopsis* transformation and molecular characterization of transgenic lines

The 35S:*PpHsp16.4*-GFP construct was introduced into *Arabidopsis* (Col-0) by *Agrobacterium*-mediated floral dip transformation method [77]. T1 seeds of infiltrated plants were collected and selected by germination on agar-solidified half strength MS medium containing 0.5% sucrose and 50 mg L^-1^ kanamycin. Subsequently, 50 kanamycin resistant seedlings were transferred into soil to produce seeds. Homozygous transgenic lines were produced from kanamycin resistant T2 seedlings and used for further analysis. Ten individual resistant lines of each construct were selected for detail molecular analysis. Expression of the transgenes and presence of the *PpHsp16.4*-GFP fusion protein was tested in 10 independent lines by RT-PCR and western blot analysis.

### Phenotypic characterization of *PpHsp16.4* knockout mutants

For osmotic stress, wild type and mutant genotypes from *P. patens* were grown for 3 weeks on cellophane overlaid BCDAT medium and thereafter transferred to 900 mM mannitol supplemented plates for 10 days. Plant survival was tested by transferring stressed colonies back to standard medium and monitored up to 17 days of recovery. For salt stress, a similar procedure was employed, but using 500 mM NaCl as a stressor. Heat stress was performed similarly, but incubating the plates at 37°C for 7 days.

Chlorophyll content, fresh weight and dry weight of plants were determined in three independent experiments using 3 plates containing 5 colonies per genotype per treatment and per time point. Dry weight was measured after incubation of individual plant colonies on cellophane discs for 16 h at 80°C. For the determination of chlorophyll content, each plant was ground up in a mortar containing 5 ml of 80% (volume in volume, v/v) acetone and the homogenized plant material was filtered to remove cell debris. Total chlorophyll was calculated as chlorophyll *a* + chlorophyll *b* (mg g^-1^ fresh weight) using the following formula: Chl*a* mg g^-1^ = [(12.7 × Abs663) – (2.6 × Abs645)] × ml acetone mg^-1^ fresh tissue; Chl*b* mg g^-1^ = [(22.9 × Abs645) – (4.68 × Abs663)] × ml acetone mg^-1^ fresh tissue.

### Subcellular localization of PpHsp16.4-GFP in tobacco and *Arabidopsis*

For transient expression experiments, tobacco leaf protoplasts were obtained from *in vitro* grown plants and electroporated with the construct 35S:*PpHsp16.4*-GFP or 35S:GFP, following the procedure described in [78]. Protoplasts were analyzed 24 hours after transfection by confocal laser microscopy. For agroinfiltration experiments, soil-grown tobacco plants were infiltrated with *Agrobacterium tumefaciens* cultures containing the construct 35S:*PpHsp16.4*-GFP as described [79]. Two days after infiltration, tobacco leaf squares were mounted in tap water and analyzed by confocal laser microscopy for GFP fluorescence.

Stable *Arabidopsis* transgenic lines, overexpressing *PpHsp16.4*-GFP were grown *in vitro* for 6 days and root and hypocotyl sections were mounted in tap water and analyzed by confocal microscopy. Confocal imaging was performed using a confocal laser scanning microscope Leica TCS-SP5, with an excitation of 488 nm for GFP.

### Spatial and subcellular localization of PpHsp16.4:Citrine proteins in transgenic knock-in *P. patens*

Twenty 20 days old colonies of transgenic *P. patens* lines, expressing PpHsp16.4-Citrine, under the control of the native promoter of the target gene, were used to examine the expression and localization pattern of the fusion protein in protonema filaments and in the leafy gametophyte. Stress treatments were done for 24 hours by transferring plants to 500 mM mannitol supplemented media for osmotic stress, or by incubating them at 37°C. Protoplasts from *P. patens* transgenic knock-in lines were obtained as described above for the generation of *P. patens* transgenic lines. *In vivo* fluorescence microscopic observations were made using confocal laser scanning microscope Leica TCS-SP5, using 488 nm for the fluorescence excitation of Citrine.

## Competing interest

The authors declare no competing interests.

## Authors’ contribution

CR conducted most experiments. AC participated in *P. patens* transformation and performed phylogenetical analysis. VC generated the subtracted library. LSZ contributed in the generation of *Arabidopsis* transgenic lines and the subcellular localization studies and revised the manuscript. SV designed and supervised the study, contributed to the analysis of the data and wrote the manuscript. All authors read and approved the final manuscript.

## Supplementary Material

Additional file 1 **Expression profile of *P. patens sHsp* genes in different developmental stages.** Transcript levels of *sHsp* genes are presented as heat maps generated at Genevestigator based on microarray data. Values are log-scaled to the expression potential of each gene. Click here for file

Additional file 2 **Temporal induction pattern of *PpHsp16.4.*** Total RNA samples from untreated *P. patens* wild-type plants (Ctrl) or treated with 100 μM H_2_O_2,_ 100 μM methyl viologen (MV), 500 mM Mannitol (Mtl), 300 mM NaCl, or 50 μM ABA for 4 and 48 hours (h) were analyzed by Northern blot using a ^32^P-labeled hybridization probe corresponding to the full-length cDNA sequence of *PpHsp16.4.* Ethidium bromide staining of ribosomal RNA (rRNA) was used to ensure equal loading of RNA samples. Click here for file

## References

[B1] Chinnusamy V Schumaker K Zhu JK Molecular genetic perspectives on cross-talk and specificity in abiotic stress signalling in plants J Exp Bot 2004 55 225 236 14673035 10.1093/jxb/erh005

[B2] Oliver MJ Velten J Mishler BD Desiccation tolerance in bryophytes: a reflection of the primitive strategy for plant survival in dehydrating habitats? Integr Comp Biol 2005 45 788 799 10.1093/icb/45.5.788 21676830

[B3] Shinozaki K Yamaguchi-Shinozaki K Molecular responses to dehydration and low temperature: differences and cross-talk between two stress signaling pathways Curr Opin Plant Biol 2000 3 217 223 10837265

[B4] Bray EA Plant responses to water deficit Trends Plant Sci 1997 2 48 54

[B5] Zhu JK Salt and drought stress signal transduction in plants Annu Rev Plant Biol 2002 53 247 273 10.1146/annurev.arplant.53.091401.143329 12221975 PMC3128348

[B6] Bouchabke-Coussa O Quashie M-L Seoane-Redondo J Fortabat M-N Gery C Yu A Linderme D Trouverie J Granier F Teoule E Durand-Tardif M ESKIMO1 is a key gene involved in water economy as well as cold acclimation and salt tolerance BMC Plant Biol 2008 8 125 10.1186/1471-2229-8-125 19061521 PMC2630945

[B7] Krasensky J Jonak C Drought, salt, and temperature stress-induced metabolic rearrangements and regulatory networks J Exp Bot 2012 63 1593 1608 10.1093/jxb/err460 22291134 PMC4359903

[B8] Scharf KD Siddique M Vierling E The expanding family of *Arabidopsis thaliana* small heat stress proteins and a new family of proteins containing alpha-crystallin domains (Acd proteins) Cell Stress Chaperon 2001 6 225 237 10.1379/1466-1268(2001)006<0225:TEFOAT>2.0.CO;2 PMC434404 11599564

[B9] Forreiter C Kirschner M Nover L Stable transformation of an *Arabidopsis* cell suspension culture with firefly luciferase providing a cellular system for analysis of chaperone activity in vivo Plant Cell 1997 9 2171 2181 9437862 10.1105/tpc.9.12.2171 PMC157066

[B10] Lee GJ Roseman AM Saibil HR Vierling E A small heat shock protein stably binds heat-denatured model substrates and can maintain a substrate in a folding-competent state EMBO J 1997 16 659 671 10.1093/emboj/16.3.659 9034347 PMC1169668

[B11] Heckathorn SA Downs CA Sharkey TD Coleman JS The small, methionine-rich chloroplast heat-shock protein protects photosystem II electron transport during heat stress Plant Physiol 1998 116 439 444 10.1104/pp.116.1.439 9449851 PMC35186

[B12] Löw D Brandle K Nover L Forreiter C Cytosolic heat-stress proteins Hsp17.7 class I and Hsp17.3 class II of tomato act as molecular chaperones in vivo Planta 2000 211 575 582 10.1007/s004250000315 11030557

[B13] de Jong WW Caspers GJ Leunissen JA Genealogy of the alpha-crystallin–small heat-shock protein superfamily Int J Biol Macromol 1998 22 151 162 10.1016/S0141-8130(98)00013-0 9650070

[B14] Bondino HG Valle EM Ten Have A Evolution and functional diversification of the small heat shock protein/α-crystallin family in higher plants Planta 2012 235 1299 1313 10.1007/s00425-011-1575-9 22210597

[B15] Waters ER The evolution, function, structure, and expression of the plant sHSPs J Exp Bot 2013 64 391 403 10.1093/jxb/ers355 23255280

[B16] Sabehat A Lurie S Weiss D Expression of small heat-shock proteins at low temperatures. A possible role in protecting against chilling injuries Plant Physiol 1998 117 651 658 10.1104/pp.117.2.651 9625718 PMC34985

[B17] Asada K Foyer CH, Mullineaux PM Production and action of active oxygen species in photosynthetic tissues Causes of photooxidative stress and amelioration of defense systems in plants 1994 Florida: CRC Press 77 104

[B18] Almoguera C Coca MA Jordano J Tissue-specific expression of sunflower heat shock proteins in response to water stress Plant J 1993 4 947 958 10.1046/j.1365-313X.1993.04060947.x

[B19] Alamillo J Almoguera C Bartels D Jordano J Constitutive expression of small heat shock proteins in vegetative tissues of the resurrection plant *Craterostigma plantagineum* Plant Mol Biol 1995 29 1093 1099 10.1007/BF00014981 8555452

[B20] Harndahl U Hall RB Osteryoung KW Vierling E Bornman JF Sundby C The chloroplast small heat shock protein undergoes oxidation-dependent conformational changes and may protect plants from oxidative stress Cell Stress Chaperon 1999 4 129 138 10.1379/1466-1268(1999)004<0129:TCSHSP>2.3.CO;2 PMC312927 10547062

[B21] Hamilton EW Heckathorn SA Mitochondrial adaptations to NaCl. Complex I is protected by anti-oxidants and small heat shock proteins, whereas complex II is protected by proline and betaine Plant Physiol 2001 126 1266 1274 10.1104/pp.126.3.1266 11457977 PMC116483

[B22] Sun W Van Montagu M Verbruggen N Small heat shock proteins and stress tolerance in plants Biochim Biophys Acta 2002 1577 1 9 10.1016/S0167-4781(02)00417-7 12151089

[B23] Wehmeyer N Vierling E The expression of small heat shock proteins in seeds responds to discrete developmental signals and suggests a general protective role in desiccation tolerance Plant Physiol 2000 122 1099 1108 10.1104/pp.122.4.1099 10759505 PMC58944

[B24] Wu C Heat shock transcription factors: structure and regulation Annu Rev Cell Develop Biol 1995 11 441 469 10.1146/annurev.cb.11.110195.002301 8689565

[B25] Schöffl F Prändl R Reindl A Regulation of the heat-shock response Plant Physiol 1998 117 1135 1141 10.1104/pp.117.4.1135 9701569 PMC1539185

[B26] Pelham HR A regulatory upstream promoter element in the *Drosophila* hsp 70 heat-shock gene Cell 1982 30 517 528 10.1016/0092-8674(82)90249-5 6814763

[B27] von Koskull-Doring P Scharf K-D Nover L The diversity of plant heat stress transcription factors Trends Plant Sci 2007 12 452 457 10.1016/j.tplants.2007.08.014 17826296

[B28] Kotak S Vierling E Bäumlein H Koskull-Döring PV A novel transcriptional cascade regulating expression of heat stress proteins during seed development of *Arabidopsis* Plant Cell 2007 19 182 195 10.1105/tpc.106.048165 17220197 PMC1820961

[B29] Ehrnsperger M Graber S Gaestel M Buchner J Binding of non-native protein to Hsp25 during heat shock creates a reservoir of folding intermediates for reactivation EMBO J 1997 16 221 229 10.1093/emboj/16.2.221 9029143 PMC1169629

[B30] Veinger L Diamant S Buchner J Goloubinoff P The small heat-shock protein IbpB from Escherichia coli stabilizes stress-denatured proteins for subsequent refolding by a multichaperone network J Biol Chem 1998 273 11032 11037 10.1074/jbc.273.18.11032 9556585

[B31] Lee GJ Vierling E A small heat shock protein cooperates with heat shock protein 70 systems to reactivate a heat-denatured protein Plant Physiol 2000 122 189 198 10.1104/pp.122.1.189 10631262 PMC58857

[B32] Reddy GB Das KP Petrash JM Surewicz WK Temperature-dependent chaperone activity and structural properties of human alphaA- and alphaB-crystallins J Biol Chem 2000 275 4565 4570 10.1074/jbc.275.7.4565 10671481

[B33] Nakamoto H Vigh L The small heat shock proteins and their clients Cell Mol Life Sci 2007 64 294 306 10.1007/s00018-006-6321-2 17187175 PMC11138444

[B34] Waters ER Lee GJ Vierling E Evolution, structure and function of the small heat shock proteins in plants J Exp Bot 1996 47 325 338 10.1093/jxb/47.3.325

[B35] Mogk A Schlieker C Friedrich KL Schonfeld HJ Vierling E Bukau B Refolding of substrates bound to small Hsps relies on a disaggregation reaction mediated most efficiently by ClpB/DnaK J Bioll Chem 2003 278 31033 31042 10.1074/jbc.M303587200 12788951

[B36] Schaefer DG Zryd JP Efficient gene targeting in the moss *Physcomitrella patens* Plant J 1997 11 1195 1206 10.1046/j.1365-313X.1997.11061195.x 9225463

[B37] Saavedra L Svensson J Carballo V Izmendi D Welin B Vidal S A dehydrin gene in *Physcomitrella patens* is required for salt and osmotic stress tolerance Plant J 2006 45 237 249 10.1111/j.1365-313X.2005.02603.x 16367967

[B38] Charron AJ Quatrano RS Between a rock and a dry place: the water-stressed moss Mol Plant 2009 2 478 486 10.1093/mp/ssp018 19825631

[B39] Frank W Ratnadewi D Reski R *Physcomitrella patens* is highly tolerant against drought, salt and osmotic stress Planta 2005 220 384 394 10.1007/s00425-004-1351-1 15322883

[B40] Wang XQ Yang PF Liu Z Liu WZ Hu Y Chen H Kuang TY Pei ZM Shen SH He YK Exploring the mechanism of *Physcomitrella patens* desiccation tolerance through a proteomic strategy Plant Physiol 2009 149 1739 1750 10.1104/pp.108.131714 19211702 PMC2663739

[B41] Goodstein DM Shu S Howson R Neupane R Hayes RD Fazo J Mitros T Dirks W Hellsten U Putnam N Phytozome: a comparative platform for green plant genomics Nucleic Acids Res 2012 40 D1178 D1186 10.1093/nar/gkr944 22110026 PMC3245001

[B42] Thompson JD Gibson TJ Plewniak F Jeanmougin F Higgins DG The CLUSTAL_X windows interface: flexible strategies for multiple sequence alignment aided by quality analysis tools Nucleic Acids Res 1997 25 4876 4882 10.1093/nar/25.24.4876 9396791 PMC147148

[B43] Tamura K Peterson D Peterson N Stecher G Nei M Kumar S MEGA5: molecular evolutionary genetics analysis using maximum likelihood, evolutionary distance, and maximum parsimony methods Mol Biol Evol 2011 28 2731 2739 10.1093/molbev/msr121 21546353 PMC3203626

[B44] Zimmermann P Laule O Schmitz J Hruz T Bleuler S Gruissem W Genevestigator transcriptome meta-analysis and biomarker search using rice and barley gene expression databases Mol Plant 2008 1 851 857 10.1093/mp/ssn048 19825587

[B45] Ruibal C Salomó Pérez I Carballo V Castro A Bentancor M Borsani O Szabados L Vidal S Differential contribution of individual dehydrin genes from *Physcomitrella patens* to salt and osmotic stress tolerance Plant Sci 2012 190 89 102 22608523 10.1016/j.plantsci.2012.03.009

[B46] Khandelwal A Cho SH Marella H Sakata Y Perroud PF Pan A Quatrano RS Role of ABA and ABI3 in desiccation tolerance Science 2010 327 546 10.1126/science.1183672 20110497

[B47] Heikal AA Hess ST Baird GS Tsien RY Webb WW Molecular spectroscopy and dynamics of intrinsically fluorescent proteins: coral red (dsRed) and yellow (Citrine) Proc Natl Acad Sci USA 2000 97 11996 12001 10.1073/pnas.97.22.11996 11050231 PMC17283

[B48] Kamisugi Y Schlink K Rensing SA Schween G von Stackelberg M Cuming AC Reski R Cove DJ The mechanism of gene targeting in *Physcomitrella patens*: homologous recombination, concatenation and multiple integration Nucleic Acids Res 2006 34 6205 6214 10.1093/nar/gkl832 17090599 PMC1693892

[B49] Timperio AM Egidi MG Zolla L Proteomics applied on plant abiotic stresses: role of heat shock proteins (HSP) J proteomics 2008 71 391 411 10.1016/j.jprot.2008.07.005 18718564

[B50] Swindell W Huebner M Weber A Transcriptional profiling of *Arabidopsis* heat shock proteins and transcription factors reveals extensive overlap between heat and non-heat stress response pathways BMC Genomics 2007 8 125 10.1186/1471-2164-8-125 17519032 PMC1887538

[B51] Siddique M Gernhard S von Koskull-Doring P Vierling E Scharf K-D The plant sHSP superfamily: five new members in *Arabidopsis thaliana* with unexpected properties Cell Stress Chaperon 2008 1 183 197 10.1007/s12192-008-0032-6 PMC2673886 18369739

[B52] Waters E Aevermann B Sanders-Reed Z Comparative analysis of the small heat shock proteins in three angiosperm genomes identifies new subfamilies and reveals diverse evolutionary patterns Cell Stress Chaperon 2008 13 127 142 10.1007/s12192-008-0023-7 PMC2673885 18759000

[B53] Scarpeci TE Zanor MI Carrillo N Mueller-Roeber B Valle EM Generation of superoxide anion in chloroplasts of *Arabidopsis thaliana* during active photosynthesis: a focus on rapidly induced genes Plant Mol Biol 2008 66 361 378 10.1007/s11103-007-9274-4 18158584 PMC2758387

[B54] Mittler R Vanderauwera S Gollery M Van Breusegem F Reactive oxygen gene network of plants Trends Plant Sci 2004 9 490 498 10.1016/j.tplants.2004.08.009 15465684

[B55] Miller G Mittler R Could heat shock transcription factors function as hydrogen peroxide sensors in plants? Ann Bot 2006 98 279 288 10.1093/aob/mcl107 16740587 PMC2803459

[B56] Neta-Sharir I Isaacson T Lurie S Weiss D Dual role for tomato heat shock protein 21: protecting photosystem II from oxidative stress and promoting color changes during fruit maturation Plant Cell 2005 17 1829 1838 10.1105/tpc.105.031914 15879560 PMC1143080

[B57] Banzet N Richaud C Deveaux Y Kazmaier M Gagnon J Triantaphylides C Accumulation of small heat shock proteins, including mitochondrial HSP22, induced by oxidative stress and adaptive response in tomato cells Plant J 1998 13 519 527 10.1046/j.1365-313X.1998.00056.x 9680997

[B58] Larkindale J Huang B Thermotolerance and antioxidant systems in *Agrostis stolonifera*: involvement of salicylic acid, abscisic acid, calcium, hydrogen peroxide, and ethylene J Plant Physiol 2004 161 405 413 10.1078/0176-1617-01239 15128028

[B59] Larkindale J Hall JD Knight MR Vierling E Heat stress phenotypes of *Arabidopsis* mutants implicate multiple signaling pathways in the acquisition of thermotolerance Plant Physiol 2005 138 882 897 10.1104/pp.105.062257 15923322 PMC1150405

[B60] Clarke SM Mur LAJ Wood JE Scott IM Salicylic acid dependent signaling promotes basal thermotolerance but is not essential for acquired thermotolerance in *Arabidopsis thaliana* Plant J 2004 38 432 447 10.1111/j.1365-313X.2004.02054.x 15086804

[B61] Chang P-FL JT-L Huang W-K Chen Y Chang H-M Wang C-W Induction of a cDNA clone from rice encoding a class II small heat shock protein by heat stress, mechanical injury, and salicylic acid Plant Sci 2007 172 64 75 10.1016/j.plantsci.2006.07.017

[B62] Saidi Y Domini M Choy F Zryd JP Schwitzguebel JP Goloubinoff P Activation of the heat shock response in plants by chlorophenols: transgenic *Physcomitrella patens* as a sensitive biosensor for organic pollutants Plant Cell Environ 2007 30 753 763 10.1111/j.1365-3040.2007.01664.x 17470151

[B63] Liu H-T Liu Y-Y Pan Q-H Yang H-R Zhan J-C Huang W-D Novel interrelationship between salicylic acid, abscisic acid, and PIP2-specific phospholipase C in heat acclimation-induced thermotolerance in pea leaves J Exp Bot 2006 57 3337 3347 10.1093/jxb/erl098 16908502

[B64] Kotak S Larkindale J Lee U von Koskull-Doring P Vierling E Scharf K-D Complexity of the heat stress response in plants Curr Opin Plant Biol 2007 10 310 316 10.1016/j.pbi.2007.04.011 17482504

[B65] Sarkar N Kim Y-K Grover A Rice sHsp genes: genomic organization and expression profiling under stress and development BMC Genomics 2009 10 393 10.1186/1471-2164-10-393 19703271 PMC2746236

[B66] Powles SB Photoinhibition of photosynthesis induced by visible light Annu Rev Plant Physiol 1984 35 15 44 10.1146/annurev.pp.35.060184.000311

[B67] Kim DH Xu Z-Y Na YJ Yoo Y-J Lee J Sohn E-J Hwang I Small heat shock protein Hsp17.8 functions as an AKR2A cofactor in the targeting of chloroplast outer membrane proteins in *Arabidopsis* Plant Physiol 2011 157 132 146 10.1104/pp.111.178681 21730198 PMC3165864

[B68] Baldwin AJ Lioe H Robinson CV Kay LE Benesch JL alphaB-crystallin polydispersity is a consequence of unbiased quaternary dynamics J Mol Biol 2011 413 297 309 10.1016/j.jmb.2011.07.016 21839090

[B69] Schaefer D Zryd JP Knight CD Cove DJ Stable transformation of the moss *Physcomitrella patens* Mol Gen Genet 1991 226 418 424 2038304 10.1007/BF00260654

[B70] Ashton NW Cove DJ The isolation and preliminary characterisation of auxotrophic and analogue resistant mutants of the moss, *Physcomitrella patens* Molec Gen Genet 1977 154 87 95 10.1007/BF00265581

[B71] Maliga PBSZ Marton L Streptomycin resistant plants from callus culture of haploid tobacco Nature 1973 244 29 30 10.1038/newbio244029a0 4515911

[B72] Murashige R Skoog F A revised medium for rapid growth and bioassays with tobacco tissue cultures Physiol Plant 1962 15 473 497 10.1111/j.1399-3054.1962.tb08052.x

[B73] Sambrook J Fritsch EF Maniatis TA Molecular Cloning: A Laboratory Manual 1989 2 Cold Spring Harbor NY: Cold Spring Harbor Laboratory Press

[B74] Dellaporta S Wood J Hicks J A plant DNA minipreparation: Version II Plant Mol Biol Rep 1983 1 19 21 10.1007/BF02712670

[B75] Karimi M Inze D Depicker A GATEWAY vectors for *Agrobacterium*-mediated plant transformation Trends Plant Sci 2002 7 193 195 10.1016/S1360-1385(02)02251-3 11992820

[B76] Koncz C Schell J The promoter of TL-DNA gene 5 controls the tissue-specific expression of chimaeric genes carried by a novel type of *Agrobacterium* binary vector Molec Gen Genet 1986 204 383 396 10.1007/BF00331014

[B77] Clough SJ Bent AF Floral dip: a simplified method for *Agrobacterium*-mediated transformation of *Arabidopsis thaliana* Plant J 1998 16 735 743 10.1046/j.1365-313x.1998.00343.x 10069079

[B78] Foresti O Da Silva LL Denecke J Overexpression of the *Arabidopsis* syntaxin PEP12/SYP21 inhibits transport from the prevacuolar compartment to the lytic vacuole in vivo Plant Cell 2006 18 2275 2293 10.1105/tpc.105.040279 16935987 PMC1560924

[B79] Neuhaus J-M Boevink P Hawes C, Satiat-Jeunemaitre B **The green fluorescent protein (GFP) as a reporter in plant cells** Plant Cell Biology 2001 Oxford, UK: Oxford University Press 127 142

