# Supplementary figures and images for: Recovery from heat, salt and osmotic stress in Physcomitrella patens requires a functional small heat shock protein PpHsp16.4
Source: BMC Plant Biol. 2013 Nov 5;13:174. doi: 10.1186/1471-2229-13-174 (PMC4228350; doi:10.1186/1471-2229-13-174)

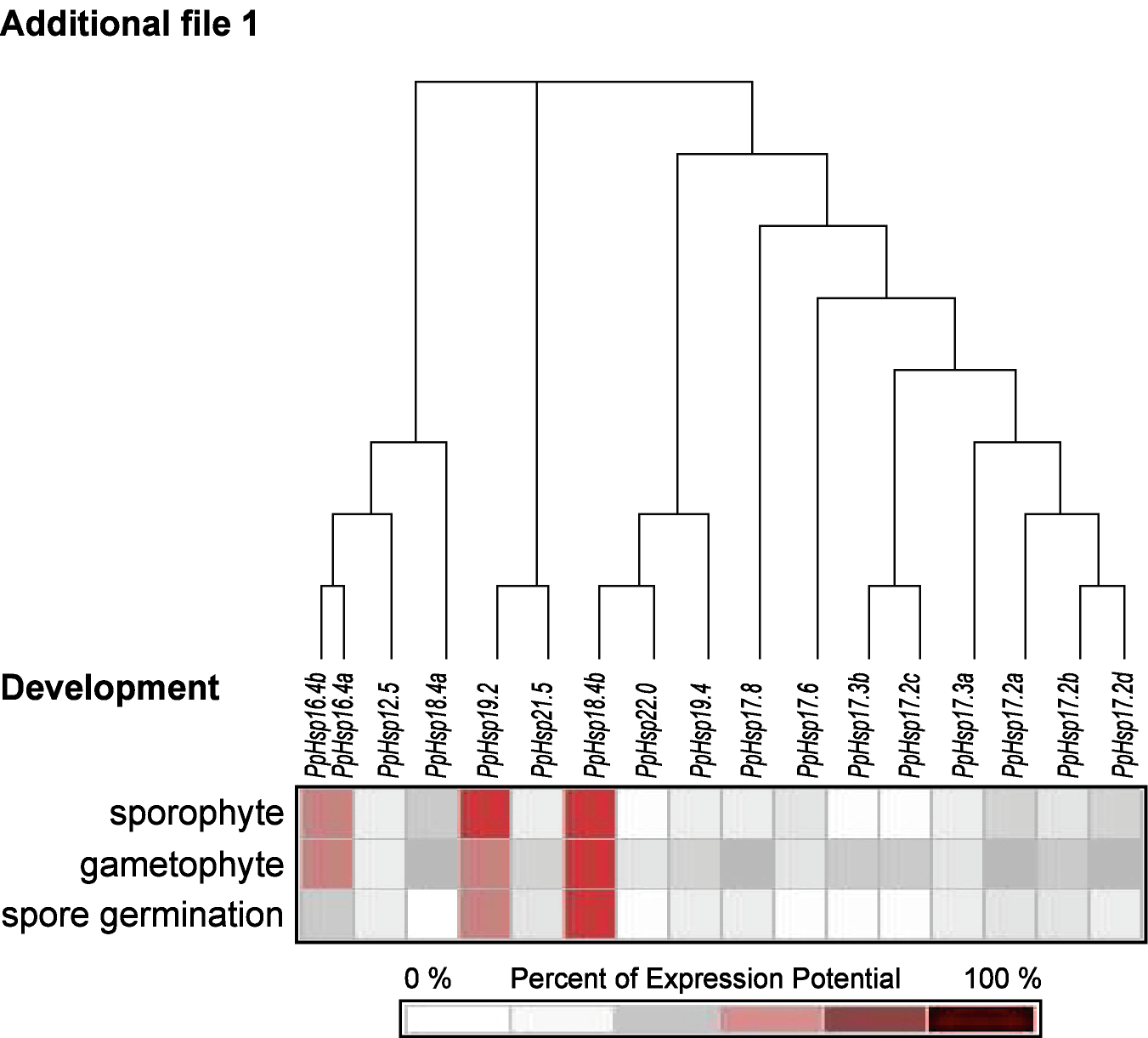

Supplement: Additional file 1 — Expression profile of P. patens sHsp genes in different developmental stages. Transcript levels of sHsp genes are presented as heat maps generated at Genevestigator based on microarray data. Values are log-scaled to the expression potential of each gene. [file 1471-2229-13-174-S1.bmp]

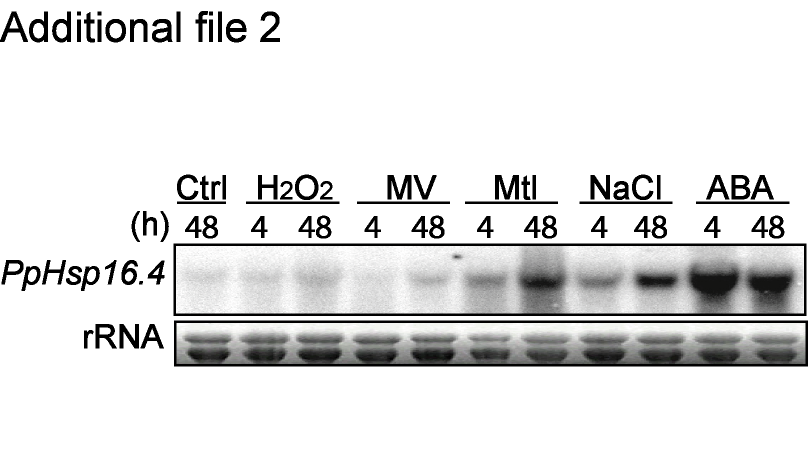

Supplement: Additional file 2 — Temporal induction pattern of PpHsp16.4. Total RNA samples from untreated P. patens wild-type plants (Ctrl) or treated with 100 μM H2O2, 100 μM methyl viologen (MV), 500 mM Mannitol (Mtl), 300 mM NaCl, or 50 μM ABA for 4 and 48 hours (h) were analyzed by Northern blot using a 32P-labeled hybridization probe corresponding to the full-length cDNA sequence of PpHsp16.4. Ethidium bromide staining of ribosomal RNA (rRNA) was used to ensure equal loading of RNA samples. [file 1471-2229-13-174-S2.bmp]
